# Supplementary material for: Predicting visual function by interpreting a neuronal wiring diagram
Source: Nature. 2024 Oct 2;634(8032):113–23. doi: 10.1038/s41586-024-07953-5 (PMC11446822; doi:10.1038/s41586-024-07953-5)
Supplement: Supplementary file 6 — CRF and ERF predictions for individual TmY4 and TmY9 cells. Analogous to Supplementary Data 3, but for TmY target types. Shown are the top four monosynaptic pathways, the strongest pathway passing through each of the top ten intermediary types (ranking from Extended Data Fig. 7), and the trisynaptic pathway Tm1–TmY–Dm3–TmY (see the section entitled Prediction of spatial normalization). [file 41586_2024_7953_MOESM6_ESM.zip › DataS4/TmY9qΓèÑ/720575940625533884.pdf]

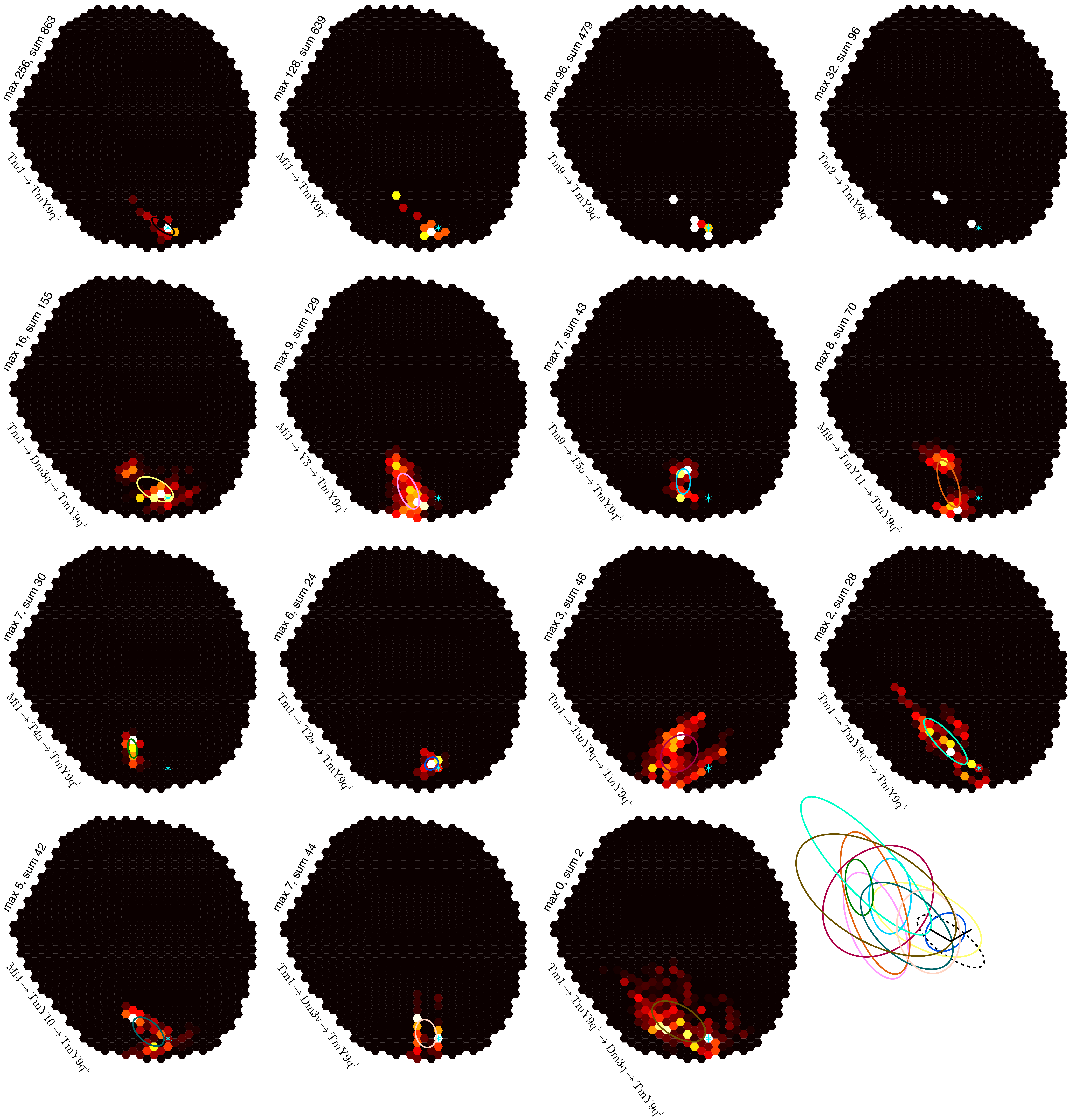

max 256, sum 863
Tm1 → TmY9q⊥
max 128, sum 639
Mi1 → TmY9q⊥
max 96, sum 479
Tm9 → TmY9q⊥
max 32, sum 96
Tm2 → TmY9q⊥
max 16, sum 155
Tm1 → Dm3q → TmY9q⊥
max 9, sum 129
Mi1 → Y3 → TmY9q⊥
max 7, sum 43
Tm9 → T5a → TmY9q⊥
max 8, sum 70
Mi9 → TmY11 → TmY9q⊥
max 7, sum 30
Mi1 → T4a → TmY9q⊥
max 6, sum 24
Tm1 → T2a → TmY9q⊥
max 3, sum 46
Tm1 → TmY9q → TmY9q⊥
max 2, sum 28
Tm1 → TmY9q⊥ → TmY9q⊥
max 5, sum 42
Mi4 → TmY10 → TmY9q⊥
max 7, sum 44
Tm1 → Dm3v → TmY9q⊥
max 0, sum 2
Tm1 → TmY9q⊥ → Dm3q → TmY9q⊥
